# Synergistic Effects of Catalyst Mixtures on Biomass Catalytic Pyrolysis

**DOI:** 10.3389/fbioe.2020.615134

**Published:** 2020-12-14

**Authors:** Badr A. Mohamed, Naoko Ellis, Chang Soo Kim, Xiaotao Bi

**Affiliations:** ^1^Department of Agricultural Engineering, Cairo University, Giza, Egypt; ^2^Department of Chemical and Biological Engineering, University of British Columbia, Vancouver, BC, Canada; ^3^Clean Energy Research Center, Korea Institute of Science and Technology, Seoul, South Korea

**Keywords:** synergistic effects of catalysts, microwave catalytic pyrolysis, K_3_PO_4_, clinoptilolite, bentonite, biomass pyrolysis

## Abstract

This paper studied the synergistic effects of catalyst mixtures on biomass catalytic pyrolysis in comparison with the single catalyst in a microwave reactor and a TGA. In general, positive synergistic effects were identified based on increased mass loss rate, reduced activation energy, and improved bio-oil quality compared to the case with a single catalyst at higher catalyst loads. 10KP/10Bento (a mixture of 10% K_3_PO_4_ and 10% bentonite) increased the mass loss rate by 85 and 45% at heating rates of 100 and 25°C/min, respectively, compared to switchgrass without catalyst. The activation energy for 10KP/10Bento and 10KP/10Clino (a mixture of 10% K_3_PO_4_ and 10% clinoptilolite) was slightly lower or similar to other catalysts at 30 wt.% load. The reduction in the activation energy by the catalyst mixture was higher at 100°C/min than 25°C/min due to the improved catalytic activity at higher heating rates. Synergistic effects are also reflected in the improved properties of bio-oil, as acids, aldehydes, and anhydrosugars were significantly decreased, whereas phenol and aromatic compounds were substantially increased. 30KP (30% K_3_PO_4_) and 10KP/10Bento increased the content of alkylated phenols by 341 and 207%, respectively, in comparison with switchgrass without catalyst. Finally, the use of catalyst mixtures improved the catalytic performance markedly, which shows the potential to reduce the production cost of bio-oil and biochar from microwave catalytic pyrolysis.

## Introduction

The global energy demand is heavily relied on fossil fuels, with more than 80% of the world’s supply of primary energy from fossil fuel in 2019. The concerns on the rising environmental impacts call for clean and renewable energy sources, including biomass energy (Chandrasekaran and Hopke, 2012; Rezaei et al., 2014; Bundhoo, 2018; Yang et al., 2019). Biomass can be converted either biologically or thermochemically to biofuels in solid, liquid, or gaseous forms. In fast pyrolysis, biomass is decomposed under oxygen-free environment into multiple products, including biochar, bio-oil, and non-condensable gases, over a very short reaction time (Chandrasekaran and Hopke, 2012; Rezaei et al., 2014; Wang et al., 2019; Sun et al., 2020; Yu et al., 2020). The challenge remains on how to upgrade pyrolysis bio-oil to drop-in liquid fuels due to their high oxygen content, high viscosity, and acidity. Several techniques have been investigated, including the use of catalytic materials and microwave heating, to enhance the quality of bio-oil and biochar (Budarin et al., 2009; Luque et al., 2012; Bundhoo, 2018; State et al., 2019; Hua et al., 2020). Catalytic fast pyrolysis has been found to be effective in improving the quality of bio-oil (Carlson et al., 2011; Rezaei et al., 2014; Bundhoo, 2018; Song et al., 2020; Yu et al., 2020). Given that high oxygen content of bio-oil can contribute to high acidity, viscosity, and reduced heating value of bio-oil, deoxygenation is essential for improving bio-oil quality and stability. Deoxygenation includes demethoxylation, decarboxylation, decarbonylation, and dehydration (Oh et al., 2017; Bundhoo, 2018; State et al., 2019; Song et al., 2020). Hydrocracking, dehydration, hydrogenation, and deoxygenation can further enhance the quality of bio-oil, in terms of acidity, heating value, and thermal stability (Oh et al., 2017; State et al., 2019; Sun et al., 2020). Catalytic pyrolysis has also been extensively investigated, using many catalysts such as natural and synthetic zeolites (Fe-H-ZSM-5-IE, ZSM-5, and H-MORD-20-IE) and natural clays (clinoptilolite, mergel, kaoline, sepiolite, and attapulgite) (Pütün et al., 2006; Pütun et al., 2009; Sulman et al., 2009; Veses et al., 2015).

Microwave-assisted pyrolysis has been proposed as an alternative heating method to increase the heating rate, thereby improving the quality of bio-oil, biochar, and syngas (Miura et al., 2004; Budarin et al., 2009; Wan et al., 2009; Lin et al., 2012; State et al., 2019; Yu et al., 2020). It is found that biochar generated from microwave-assisted pyrolysis has a greater specific surface area and pore volume than obtained from conventional fast pyrolysis because of the unique heating mechanism of microwave radiation, in which particles are directly heated (Domínguez et al., 2007; Luque et al., 2012; Yu et al., 2020). Tripotassium phosphate (K_3_PO_4_), clinoptilolite, and bentonite, which were previously investigated for catalytic pyrolysis, showed improvements in bio-oil quality as reflected by the decreased acidity, viscosity, and the increased fractions of aromatics and hydrocarbons, which are beneficial for the production of liquid biofuels (Pütun et al., 2009; Lu et al., 2013; Veses et al., 2015; Mohamed et al., 2016a, 2019a). In addition, those catalysts also improved the quality of the produced biochar with increased cation exchange capacity (CEC), surface area, and nutrient content (Satje and Nelson, 2009; Mohamed et al., 2016a). Overall, it has been found that biochars produced from mixing K_3_PO_4_ and clinoptilolite or bentonite with biomass showed effectiveness in reducing toxicity and the uptake of heavy metals (i.e., Pb, Ni, and Co) due to the predominance of different immobilization mechanisms and increased crop productivity in soils contaminated with heavy metals (Mohamed et al., 2017).

Mass and heat transfer are known to play an important role in determining the distribution of pyrolysis components and their performance by affecting both the primary decomposition reactions and the secondary reactions of primary vapors (Uzun et al., 2007; Dupont et al., 2009). Biomass pyrolysis involves many simultaneous and continuous reactions. Fundamental studies on biomass catalytic pyrolysis kinetics will help explain the catalyst behavior and reactivity of biomass materials (Zhou et al., 2015; Song et al., 2020; Wang et al., 2020). The cracking, repolymerization, and charring reactions will increase solid yield and can significantly impact the distribution of pyrolysis products, and vapors must therefore be rapidly removed from the reaction zone to avoid secondary reactions (Uzun et al., 2007; Dupont et al., 2009). In addition, catalytic coke is formed from the oxygenated volatile intermediates and the dehydrated species (Zhang et al., 2014; Müller et al., 2015). Catalytic coke deposition on catalyst surfaces is a major concern for catalyst deactivation as it reduces catalyst activity and affects pyrolysis product distribution.

In our previous work, we studied the effects of catalyst mixtures on the microwave heating behavior and found that the type of coke (e.g., graphitic carbon, oxygenated carbon, etc.) deposited on catalyst surfaces plays a key role in microwave absorption and microwave heating in microwave catalytic pyrolysis (Mohamed et al., 2019a). We also concluded that K_3_PO_4_ possesses high microwave absorption ability and inhibits the devolatilization of hemicellulose to a great extent. This promotes the formation of oxygenated coke that affects microwave absorption and biomass decomposition remarkably, resulting in a substantial increase in the solid yield by ∼53% compared to the control case (Mohamed et al., 2019a). However, bentonite does not have an inhibitory effect on hemicellulose decomposition in our previous study despite its high thermal conductivity, although it has a poor microwave absorption capacity (Mohamed et al., 2019b). Thus, mixing the two catalysts together substantially increases microwave absorption and reduces the solid yield by reducing the formation of oxygenated coke on the catalyst surface (Mohamed et al., 2019a). In the microwave reactor, microwave absorption abilities of different catalysts and catalyst mixtures may result in different microwave heating rates, which makes it difficult to elucidate the synergistic effects of catalyst mixtures on biomass decomposition unless a temperature-programmable direct-heating microwave reactor is used, which is unfortunately unavailable. Thus, to eliminate the effects of microwave heating rate on elucidating the synergistic effects of catalyst mixtures on biomass decomposition, a TGA was used in this work to study the synergistic effects of catalyst mixtures on biomass catalytic pyrolysis reaction kinetics under similar heating rates. To understand the effects of the catalyst mixtures on the catalytic selectivity and mechanism, the chemical composition of the produced bio-oils from catalyst mixtures and single catalyst in a microwave reactor were characterized and compared with the results from the control samples without catalyst and with a single catalyst.

In our view, a knowledge gap still exists about synergistic effects on aromatization reactions, selectivity, and the deoxygenation behavior of selected catalyst mixtures in microwave-assisted pyrolysis. The aims of this study are to investigate (1) the reaction kinetics of biomass catalytic pyrolysis using catalyst mixtures; (2) the kinetics of catalytic pyrolysis of biomass component (i.e., cellulose, hemicellulose, and lignin); and (3) the deoxygenation behavior and catalytic selectivity of the selected catalysts through analyzing bio-oil properties compared to the use of a single catalyst. The findings from this research will help in the design and optimization of biomass catalytic pyrolysis microwave reactors.

## Materials and Methods

### Preparation of Biomass and Catalyst Mixtures

Switchgrass was crushed and sieved into small particles (300–600 μm). The ultimate and proximate analyses of switchgrass were carried out; the moisture, ash, volatile matter, and fixed carbon contents are 5.1, 6.3, 76.9, and 11.7 wt.%, respectively, and the higher heating value (HHV) is 19.6 MJ/kg. The C, H, N, S, and O contents were determined using a CHNS/O analyzer, Perkin-Elmer 2400Series II, and the values are 47.9, 6.2, 0.8, 0.1, and 38.7 wt.% (O content was calculated by difference), respectively. Bentonite and K_3_PO_4_ were purchased from Sigma-Aldrich, Canada Co. Clinoptilolite was purchased from Bear River Zeolite Co., United States.

### Microwave and TGA Catalytic Pyrolysis

In each test run of microwave catalytic pyrolysis, a total of 20.0 g of switchgrass (SG), or switchgrass mixed with different loads of natural zeolite (clinoptilolite), bentonite, and K_3_PO_4_, was pyrolyzed in a tubular quartz reactor. To evaluate the synergistic effect of catalyst mixtures on increasing microwave heating rate and optimizing catalytic activity, various catalyst combinations were used. Owing to the weak microwave absorption by switchgrass, SiC, a chemically inert and good microwave absorber, has been mixed with SG as the control sample. The samples of pre-mixed biomass and catalyst were loaded into the reactor and purged by nitrogen at 1.5 L/min for around 30 min to generate an anoxic state. A microwave power of 750 W was utilized to heat the sample to 400°C, and the microwave power was then decreased to maintain the temperature at constant for ∼10 min. A three-stage condensation system by cold water quench was used to condense the pyrolysis volatiles, with the bio-oil being collected from bottles connected to each condenser. The condensates adhering to the interior wall of the quartz tube reactor and the condensers were recovered by washing with ethanol. Ethanol was evaporated using a rotary vacuum evaporator at 40°C until constant weight was reached. At the end of each experiment, the microwave was turned off with nitrogen flow running to cool down the biochar to about 25°C before it is removed from the microwave reactor. More information about the microwave reactor and experimental setup can be found in our previous study (Mohamed et al., 2016b).

A thermogravimetric analysis of the samples was conducted using SDT Q600 TGA at a nitrogen flow rate of 100 ml/min. To obtain a low Biot number (<0.01) for reducing the internal heat and mass transfer, switchgrass was sieved into small particles (100–150 μm). K_3_PO_4_, bentonite, and clinoptilolite powders < 50 μm were added to the biomass sample at 30 wt.% load for single catalyst case, and 20 wt.% for catalyst mixtures (10/10 wt.%). The sample was premixed before it was loaded into a crucible made of alumina, and the weight was recorded every 0.5 s, and the accuracy of the balance is 0.0001 mg. The experiments were performed at different heating rates of 25°C and 100°C/min.

### Reaction Kinetics

Pyrolysis is a complex process involving many parallel and consecutive reactions, and usually simplified by lumped reaction kinetic models (Wang et al., 2019). The primary components of the lignocellulosic substances are cellulose, lignin, and hemicellulose. The average values for the three biomass components reported for switchgrass are 32.1, 36.0, and 25.6 wt.% for cellulose, hemicellulose, and lignin, respectively (Uzun et al., 2007). Each major component is assumed to decompose following an n*th* order reaction, with the rate constant following the Arrhenius function. A kinetic reaction model with n*th* order reaction for each of the three major components can be used to study the reaction kinetics and determine the activation energy for catalytic and non-catalytic pyrolysis, according to the following equations:

(1) $$k_{i}=A_{i}exp^{-\frac{E_{i}}{RT}}$$

(2) $$\frac{d\alpha_{i}}{dt}=k_{i}\cdot f\left(\alpha_{i}\right)$$

(3) $$\begin{array}{c} f\left(\alpha_{i}\right)={\left(1-\alpha_{i}\right)}^{n_{i}}\\ \alpha_{i}=\frac{m_{io}-m_{it}}{m_{io}-m_{if}} \end{array}$$

(4) $$\frac{d\alpha_{i}}{dt}=A_{i}\cdot \left(exp^{-\frac{E_{i}}{RT}}\right)\cdot {\left(1-\alpha_{i}\right)}^{n_{i}}$$

To make the model independent on the heating rate (β = *dT/dt*), equation 4 was divided by β and then rearranged in the following form:

(5) $$\frac{d\alpha_{i}}{dT}=\left(\frac{A_{i}}{\beta }\right)\cdot \left(exp^{-\frac{E_{i}}{RT}}\right)\cdot {\left(1-\alpha_{i}\right)}^{n_{i}}$$

(6) $$\frac{d\alpha_{j}}{dT}=\sum_{j=1}^{n}C_{j}\left(\frac{A_{j}}{\beta }\right)\cdot \left(exp^{-\frac{E_{j}}{RT}}\right)\cdot {\left(1-\alpha_{j}\right)}^{n_{j}}$$

Lignocellulosic biomass materials usually contain three main components (i.e., cellulose, hemicellulose, and lignin) that decompose at different temperatures with distinctive peaks, and the deconvoluted peaks are frequently used to study the kinetics of each pseudo-component without extracting the three components from biomass (Branca et al., 2005; Di Blasi, 2008; Vamvuka and Sfakiotakis, 2011; Zhou et al., 2015). The term “pseudo-component” is usually used to represent a group of several compounds that have similar molecular structures of different chain lengths such as lignin, cellulose, and hemicellulose, which decompose following the similar kinetics as identified in the weight loss curve (Di Blasi, 2008). The three-parallel reaction model is widely adopted to describe the reaction kinetics of lignocellulosic biomass materials, and it is assumed that the three major pseudo-components (i.e., lignin, cellulose, and hemicellulose) decompose independently (Branca et al., 2005; Di Blasi, 2008; Vamvuka and Sfakiotakis, 2011).

Coats–Redfern’s integral method has been used by many researchers to determine the kinetic parameters for catalytic and non-catalytic pyrolysis (Yu et al., 2013; Poddar et al., 2015; Wang et al., 2019). According to the Coats–Redfern integral method, the simplified equation can be derived rearranging and integrating equation 5:

(7) $$\int_{0}^{\alpha }\frac{d\alpha }{f\left(\alpha \right)}=\left(\frac{A}{\beta }\right)\cdot \int_{T_{0}}^{T}\left(exp^{-\frac{E}{RT}}\right)\cdot dT$$

(8) $$\ln\left[\frac{\text{g}\left(\alpha \right)}{T^{2}}\right]=\ln\left[\frac{AR}{\beta E}\left(1-\frac{2RT}{E}\right)\right]-\frac{E}{RT}$$

where *C*_*j*_ is referred to the initial pseudo-components of biomass: lignin, cellulose, and hemicellulose; α is the conversion ratio; *A* is the frequency factor; β is the heating rate; *E is* the activation energy; *R* is the universal gas constant; *n* is the reaction order; and g(α) is the integral forms of solid pyrolysis reaction mechanistic models. Table 1 shows the expressions for different pyrolysis reaction mechanistic models (i.e., chemical reaction and diffusion-controlled reaction), which have been used by many researchers (Yu et al., 2013; Poddar et al., 2015; Wang et al., 2019).

**TABLE 1:** Integral forms of solid pyrolysis reaction mechanistic models.

Reaction model	Reaction mechanism	*g*(α)
Chemical reaction	First-order reaction (F1)	−*ln* (1−α)
	One and half order reaction (F1.5)	2[(1 − α)^−1/2^ − 1]
	Second-order reaction (F2)	(1 − α)^−1^ − 1
	Third-order reaction (F3)	[(1 − α)^−1^ − 1]/2
Diffusion-controlled reaction	One-dimensional diffusion (1D)	α^2^
	Two-dimensional diffusion (2D)	(1 − α)*ln* (1 − α) + α
	Three-dimensional diffusion (3D)	[1 − (1 − α)^1/3^]^2^

The activation energy and pre-exponential factor can be determined using linear regressions between the left-hand side of the equation for the different pyrolysis reaction mechanistic models (8) versus $\frac{1}{T}$ (Yu et al., 2013; Poddar et al., 2015). The regression graphs for different kinetic reaction mechanisms can be found in the Supplementary Material. In order to attain a more accurate reaction model, the pyrolysis process was divided into two different stages to attain a high coefficient of determination (*R*^2^) using linear regressions. Because the peak temperature for the devolatilization of biomass components changes with the heating rate and shifts to higher temperatures with increasing heating rate. The first stage was selected from 250 to 420°C for the heating rate of 25°C/min and 280 to 450°C for 100°C/min heating rate. The second stage was selected from 420 to 550°C for 25°C/min and 450 to 550°C for 100°C/min.

A catalyst can affect the reaction mechanism, leading to a different reaction order or changing kinetic control to diffusion controlled, which need to be investigated experimentally. Using the Coats–Redfern integral method to analyze the three parallel pseudo-components reactions will help to understand the catalytic effect on the three biomass pseudo-components to identify possible catalytic mechanisms.

## Results and Discussion

### Synergistic Effects of Catalyst Mixtures on Pyrolysis of Biomass in TGA

**FIGURE 1:**
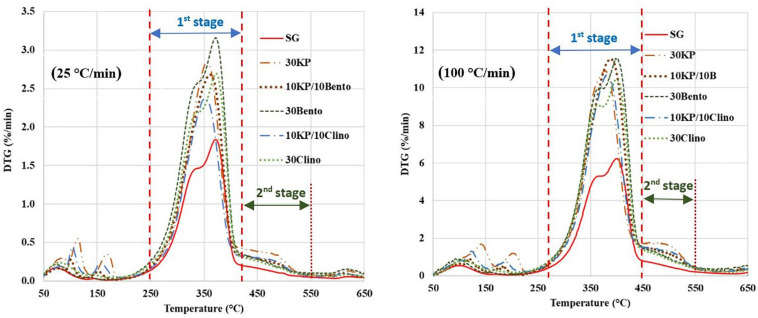
DTG curves for switchgrass and switchgrass mixed with different catalysts at heating rates of 25°C/min and 100°C/min. First stage is from 250°C to 420°C for 25°C/min and 280°C to 450°C for 100°C/min, and second stage is 450°–550°C for 25°C/min and from 450°C to 550°C at 100°C/min).

Table 2 shows the peak temperature and mass loss rate for SG and SG mixed with different catalysts. The samples containing 30 wt.% K_3_PO_4_ showed only one major peak, and the DTG peaks shifted to lower temperatures by up to 35°C from SG (Figure 1). The synergistic effects of the catalyst mixtures were observed in the mass loss rate when compared to the case with single catalyst at different heating rates. 10KP/10Bento showed the highest mass loss rate at 100°C/min, and the mass loss rate increased by 85 and 45% at 100°C/min and 25°C/min, respectively, compared to SG without the catalyst. The higher mass loss rate at higher heating rate may be related to the improved catalytic activity at higher heating rates. The lowest mass loss rate was observed for 10KP/10Clino at 25°C/min, which was significantly lower than the samples with a single catalyst at 25°C/min, but was similar to the samples with a single catalyst with 30 wt.% load at 100°C/min confirming the synergistic effects of the catalyst mixtures at 20 wt.% load. These findings confirm that the synergistic effects of the catalyst mixtures can be further enhanced by increasing the heating rate.

**TABLE 2:** Temperature (T_peak_) and mass loss rate M_peak_ (%/min) at the highest peak using two heating rates for switchgrass and switchgrass mixed with different catalysts.

	Heating rate (°C/min)	SG	30KP	30Clino	30Bento	10KP/10Clino	10KP/10Bento
T_peak_ (°C)	100	405	369	402	399	380	381
T_peak_ (°C)	25	373	354	371	371	354	356
M_peak_ (%/min)	100	6.24	11.11	10.43	11.48	10.81	11.53
M_peak_ (%/min)	25	1.83	2.85	2.69	3.16	2.37	2.75

Table 3 shows that the activation energy for 10KP/10Bento and 10KP/10Clino was approximately equal to other catalysts at 30 wt.% load. However, remarkable differences in activation energy were found at the first stage at a heating rate of 100°C/min in which most of the volatiles are released. This also agrees with the observation that synergistic effects are magnified at high heating rates. The lowest activation energy at 100°C/min was for 10KP/10Bento, which was 44 and 18% lower than those of SG and 30Bento, respectively, at the first stage. The sample with 10KP/10Clino also showed an activation energy reduction by 40 and 11% compared to those of SG and 30Clino, respectively, at the first stage. The data in the first stage of SG pyrolysis fitted well with the diffusion-controlled model “D-2” listed in Table 1 at both heating rates of 25°C/min and 100°C/min, while the chemical reaction “F-1.5” model fitted well to the second stage of SG pyrolysis. The diffusion-controlled process for SG in the first stage may correspond to the fast devolatilization of the extractives and hemicellulose compounds (Branca et al., 2005; Chandrasekaran and Hopke, 2012). The chemical reaction models of “F-1.5” and “F-1” seem to fit better with the second stage data for most of the samples at 25°C/min and 100°C/min, except 30KP where the diffusion-controlled model “F-2” gave the best fit to the second stage data at 100°C/min.

**TABLE 3:** Kinetic parameters “E (kJ/mole) and A (1/s)” at two heating rates for switchgrass and switchgrass mixed with different catalysts.

	Heating rate °C/min	SG	30KP	30Clino	30Bento	10KP/10Clino	10KP/10Bento
First stage	25 (250–420°C)	**(D-2)** *E* = 106 *A* = 2.21E+06 *R*^2^ = 0.988	**(F-1.5)** *E* = 59.7 *A* = 896 *R*^2^ = 0.975	**(F-1)** *E* = 58.1 *A* = 377 *R*^2^ = 0.986	**(F-1)** *E* = 63.1 *A* = 1002 *R*^2^ = 0.991	**(F-1)** *E* = 61.8 *A* = 130 *R*^2^ = 0.975	**(F-1)** *E* = 58.6 *A* = 395 *R*^2^ = 0.980
	100 (280–450°C)	**(D-2)** *E* = 114 *A* = 1.01E+07 *R*^2^ = 0.989	**(F-1.5)** *E* = 71.6 *A* = 18,806 *R*^2^ = 0.981	**(F-1.5)** *E* = 77.3 *A* = 50,030 *R*^2^ = 0.986	**(F-1.5)** *E* = 80.6 *A* = 89,531 *R*^2^ = 0.986	**(F-1)** *E* = 69.1 *A* = 1,083 *R*^2^ = 0.976	**(F-1)** *E* = 65.9 *A* = 3,846 *R*^2^ = 0.983
Second stage	25 (420–550°C)	**(F-1.5)** *E* = 45.9 *A* = 114 *R*^2^ = 0.999	**(F-1.5)** *E* = 31.6 *A* = 2.60 *R*^2^ = 0.990	**(F-1.5)** *E* = 23.1 *A* = 0.54 *R*^2^ = 0.999	**(F-2)** *E* = 34.4 *A* = 10.69 *R*^2^ = 0.999	**(F-1.5)** *E* = 29.6 *A* = 1.68 *R*^2^ = 0.992	**(F-1.5)** *E* = 26.1 *A* = 0.88 *R*^2^ = 0.997
	100 (450–550°C)	**(F-1.5)** *E* = 35.8 *A* = 23.7 *R*^2^ = 0.999	**(F-2)** *E* = 22.4 *A* = 0.0001 *R*^2^ = 1	**(F-1)** *E* = 28.6 *A* = 5.49 *R*^2^ = 0.999	**(F-1)** *E* = 34.9 *A* = 19.5 *R*^2^ = 0.999	**(F-1)** *E* = 16.9 *A* = 0.218 *R*^2^ = 0.993	**(F-1)** *E* = 14.6 *A* = 0.137 *R*^2^ = 0.997

Poddar et al. (2015) studied non-catalytic and catalytic pyrolysis of jute waste using zinc oxide, potassium chloride, sodium chloride, sodium aluminosilicate, and alumina as catalysts in a TGA and semi-batch pyrolyzer. It was found that alumina showed the best performance, achieving the highest bio-oil yield and lowest activation energy (Poddar et al., 2015). Song et al. (2020) investigated the catalytic effects of iron ore and iron oxide on the pyrolysis of municipal solid waste in a TGA and fixed-bed reactor, and reported that the activation energy decreased from 180 kJ/mol to ∼151 kJ/mol after using iron ore and iron oxide, with an increase in conversion rate by up to 55% for municipal solid waste pyrolysis.

### Synergistic Effects of Catalyst Mixtures on Pyrolysis of Pseudo-Components

To investigate the catalytic effects on the decomposition of the three primary biomass pseudo-components (cellulose, hemicellulose, and lignin), the conversion percentage and the activation energy for each pseudo-component were extracted by fitting the three-parallel reaction model with the TGA data. The fitted parameters describe the reaction kinetics of the three pseudo-components.

**FIGURE 2:**
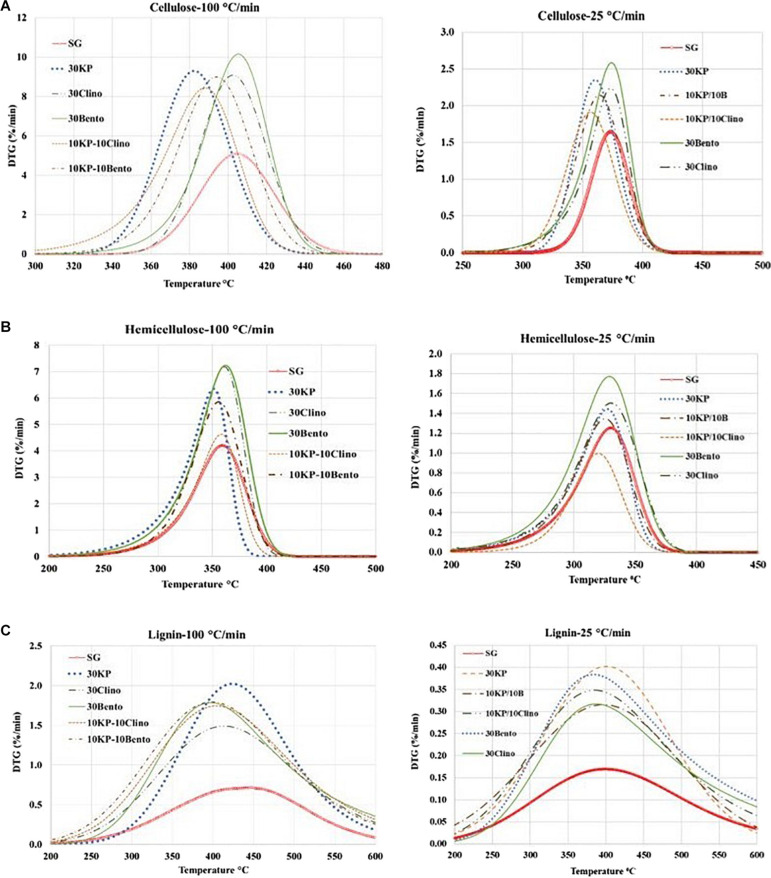
Effects of catalysts on the decomposition of **(A)** pseudo-cellulose, **(B)** pseudo-hemicellulose, and **(C)** pseudo-lignin for switchgrass and switchgrass mixed with different catalysts at heating rates of 25°C/min and 100°C/min.

Figure 2 and Table 4 show the mass loss rate of the pseudo-cellulose, pseudo-hemicellulose, and pseudo-lignin for SG with different catalysts at different heating rates. The samples that contained K_3_PO_4_ shifted the devolatilization peak of the pseudo-cellulose to lower temperatures when compared to SG and other catalysts, and a major difference was found for 30KP, at both heating rates of 25°C/min and 100°C/min. The same behavior was observed for pseudo-hemicellulose and pseudo-lignin; and the peak temperature was shifted to lower temperatures compared to SG at 25°C/min and 100°C/min (Figure 2). Comparing the samples with catalyst mixtures containing K_3_PO_4_ with bentonite or clinoptilolite, the shift in peak temperature was also observed.

**TABLE 4:** Peak temperature comparison for each biomass pseudo-component between SG and SG with different catalysts.

	Heating rate °C/min	SG	30KP	30Clino	30Bento	10KP/10Clino	10KP/10Bento
Cellulose	25	375	359	371	371	353	363
	100	407	375	400	402	385	391
Hemicellulose + extractives	25	331	325	329	328	317	321
	100	365	349	362	361	354	356
Lignin	25	416	401	389	385	388	394
	100	448	417	416	408	403	401

Table 5 shows the percentage conversion for each pseudo-component of SG, with or without catalysts, based on the solid yield between 200 and 600°C per the best fit of the experimental data. Increasing the heating rate did not affect the conversion of the three pseudo-components for SG, while different trends were observed for the samples mixed with catalysts. The highest conversion for SG was found for pseudo-cellulose, with pseudo-lignin having the lowest conversion because it is the most difficult component to decompose and thus contribute more toward final solid yield (up to 46 wt.% of its original weight) (Yang et al., 2007).

**TABLE 5:** Conversion percentage for each biomass pseudo-component (between 200 and 600°C) for SG and SG with different catalysts.

	Heating rate °C/min	SG	30KP	30Clino	30Bento	10KP/10Clino	10KP/10Bento
Cellulose	25	87.73	92.61	93.07	93.26	88.20	93.86
	100	86.62	90.47	93.69	93.69	83.63	89.82
Hemicellulose + extractives	25	82.01	65.14	88.46	91.34	45.06	69.26
	100	81.95	67.80	88.70	86.58	44.26	66.93
Lignin	25	67.20	94.34	89.10	88.68	88.93	91.52
	100	65.51	90.83	93.64	93.62	86.96	93.71

The pseudo-hemicellulose in samples containing K_3_PO_4_ showed less reactivity and had the lowest conversion (46%) for samples containing 10KP/10Clino at 25°C/min and 100°C/min. This behavior is likely caused by the inhibitory effect of K_3_PO_4_ because clinoptilolite would have increased the conversion of the pseudo-hemicellulose (Mohamed et al., 2019b). On the other hand, the highest conversion of the pseudo-lignin was observed for 30KP, which resulted in an increase of 29% in pseudo-lignin conversion compared to SG at 25°C/min. 10KP/10Bento showed the highest conversion at 100°C/min, which was 31% higher than that of the SG sample. Increasing the heating rate from 25°C/min to 100°C/min did not show any significant effects on the conversion of the three pseudo-components for all samples.

Tables 6, 7 show the kinetic parameters for the three pseudo-components at different heating rates for SG with or without catalysts. The coefficient of determination (*R*^2^) for all samples at 25°C/min and 100°C/min ranged between 0.980 and 0.999, which indicates that the kinetic models can be used to properly describe the pyrolysis reactions. The reaction order *n* ranged between 0.89 and 1.53 for all samples. The frequency factor varied widely among different heating rates and different catalysts. The activation energy of the pseudo-cellulose for SG at 25°C/min was within the range of reported values for cellulose at slow heating rates (230–250 kJ/mole) (Branca et al., 2005). The activation energy of the pseudo-cellulose for SG at 100°C/min was about 17% higher than at 25°C/min, but still fell into the range of the values reported for cellulose at high heating rates (260–285 kJ/mole) (Lin et al., 2009; Zhou et al., 2015). All catalysts reduced the activation energy of the pseudo-cellulose from the control SG sample, and the mixtures of different catalysts (20 wt.%) showed similar or lower values compared to other single catalysts at 30 wt.% load, confirming the positive synergistic effects of catalyst mixtures. The lowest value for the activation energy of the pseudo-cellulose was observed for 10KP/10Bento, with the activation energy 34 and 39% lower than the control SG sample at 25°C/min and 100°C/min, respectively. The higher reduction in activation energy at 100°C/min than at 25°C/min could be related to the improved catalytic activity at higher heating rates.

**TABLE 6:** Kinetic parameters of different pseudo-components for SG and SG with different catalysts using TGA at 25°C/min.

Pseudo-component	Kinetic parameters	SG	30KP	30Clino	30Bento	10KP/10Clino	10KP/10Bento
Cellulose	E kJ/mole	241	164	194	191	160	159
	A (1/min)	7.05E+19	3.69E+13	5.98E+15	3.59E+15	2.22E+13	9.85E+12
	*n*	1.25	1.15	1.08	1.05	1.16	1.09
	*R*^2^	(0.993)	(0.993)	(0.996)	(0.998)	(0.997)	(0.991)
Hemicellulose + extractives	E kJ/mole	105	98.1	90.2	90.2	101	99.4
	A (1/min)	1.30E+09	3.16E+08	4.51E+07	4.97E+07	5.33E+10	1.04E+09
	*n*	1.04	0.89	0.91	0.93	1.04	0.95
	*R*^2^	(0.990)	(0.989)	(0.990)	(0.992)	(0.992)	(0.995)
Lignin	E kJ/mole	47.3	37.8	38.2	38.4	34.3	32.4
	A (1/min)	1.44E+03	163	199	207	102	54.9
	*n*	1.26	0.98	1.32	1.26	1.14	0.93
	*R*^2^	(0.994)	(0.995)	(0.980)	(0.980)	(0.985)	(0.995)

**TABLE 7:** Kinetic parameters of di1fferent pseudo-components for SG and SG with different catalysts at 100°C/min.

Pseudo-component	Kinetic parameters	SG	30KP	30Clino	30Bento	10KP/10Clino	10KP/10Bento
Cellulose	E kJ/mole	281	170	230	218	176	173
	A (1/min)	3.80E+22	1.80E+14	2.26E+18	3.18E+17	3.31E+14	1.35E+13
	*n R*^2^	1.53 (0.994)	1.21 (0.999)	1.15 (0.994)	1.09 (0.994)	1.14 (0.995)	1.09 (0.995)
Hemicellulose + extractives	E kJ/mole	120	128	83	100	119	128
	A (1/min)	5.30E+09	2.85E+11	1.74E+07	4.96E+08	2.47E+10	1.19E+11
	*n R*^2^	1.09 (0.992)	1.0 (0.994)	0.89 (0.988)	0.90 (0.991)	0.98 (0.989)	0.94 (0.995)
Lignin	E kJ/mole	57	41.1	44.1	41.0	43	38.6
	A (1/min)	2.12E+04	1.32E+03	6.80E+03	1.42E+03	1.85E+03	890
	*n R*^2^	1.28 (0.997)	1.19 (0.992)	1.25 (0.988)	1.32 (0.981)	1.19 (0.989)	1.18 (0.994)

The same trends were found for the pseudo-hemicellulose of SG, and the activation energy slightly increased when the heating rate increased from 25°C/min to 100°C/min. The activation energy values were within the range of reported values for hemicellulose (Huang et al., 2011). All catalysts reduced the activation energy of the pseudo-hemicellulose significantly except the samples containing K_3_PO_4_, where the activation energy was similar to that of SG. The highest activation energy for the pseudo-hemicellulose at 25°C/min and 100°C/min was found for 10KP/10Clino. It is noted that 10KP/10Clino also gave the highest solid yield at both 25°C/min and 100°C/min, which may be related to the lower reaction rate of pseudo-hemicellulose. The obtained activation energy for pseudo-lignin of SG was similar to the reported values for lignin (Branca et al., 2005). Most of the catalysts reduced the activation energy of pseudo-lignin, with the lowest value obtained for 10KP/10Bento, which was 32% lower than that of the control SG sample. The activation energy for the pseudo-lignin of SG increased significantly as the heating rate increased from 25°C/min to 100°C/min, but the values still fell within the range of reported values for lignin (Branca et al., 2005; Huang et al., 2011). At 25°C/min, most of the catalysts reduced the activation energy of the pseudo-lignin, while the lowest value was obtained with 10KP/10Bento which was 46 and 48% lower the SG control sample at 25°C/min and 100°C/min, respectively. In summary, compared to the single catalysts, TGA test data showed that catalyst mixtures improved the pyrolysis performance by lowering the activation energy, increasing the pyrolysis reaction rate and increasing the microwave heating rate.

### Synergistic Effects of Catalyst Mixtures on Bio-Oil Properties

Bio-oil produced in the bench microwave reactor was further characterized to understand the catalyst performance in biomass pyrolysis. The chemical composition of the total bio-oil (both organic and aqueous phases) produced from SG mixed with SiC (control) and SG mixed with different catalysts and their mixtures in microwave pyrolysis is given in Table 8. Because of the poor microwave absorption, the maximum temperature of SG mixed with bentonite at different loads (i.e., 10, 20, and 30 wt.%) could not go beyond 170°C. Therefore, no bio-oil was produced from SG + bentonite. Details on bio-oil characterization can be found in our previous work (Mohamed et al., 2019a). The bio-oil chemical compositions for 30KP and 30Clino samples have been published previously and reported here for the comparison with the samples with catalyst mixtures to show their synergistic effects (Mohamed et al., 2019a, b). As shown in Table 8, catalysts changed the chemical composition of bio-oil, i.e., substantially reduced oxygenated compounds such as acids, furans and aldehydes, and significantly increased phenolic and other aromatic compounds in comparison with the control sample (SG mixed with SiC).

**TABLE 8:** Chemical composition of bio-oil (peak area %) and total bio-oil yield (wt.%) produced from SG with SiC and SG mixed with different catalysts under microwave-assisted pyrolysis.

No.	Compounds	Formula	SG + SiC	SG + 30KP	SG + 30Clino	SG + 10KP/10Bento	SG + 10KP/10Clino
y 1	2-Propanone, 1-hydroxy-	C_3_H_6_O_2_	10.14	0.50	8.81	7.83	7.30
2	2-Cyclopenten-1-one	C_5_H_6_O	1.75	0.64	2.75	0.89	0.90
3	1-Hydroxy-2-butanone	C_4_H_8_O_2_	3.00	0.89	2.42	2.34	2.22
4	Acetic acid	C_2_H_4_O_2_	21.95	4.87	16.05	11.69	17.76
5	Furfural	C_5_H_4_O_2_	3.09	–	1.36	0.83	1.62
6	Propanal, 2-methyl-	C_4_H_8_O	0.59	–	–	–	–
7	2-Propanone, 1-(acetyloxy)-	C_5_H_8_O_3_	–	0.46	–	–	–
8	2-Butanone	C_4_H_8_O	–	–	–	0.35	–
9	(3E,5E)-Hepta-3,5-dien-2-one	C_7_H_10_O	–	–	–	–	0.72
10	Propanoic acid	C_3_H_6_O_2_	2.31	1.08	1.92	1.85	0.92
11	2-Furancarboxaldehyde, 5-methyl-	C_6_H_6_O_2_	0.52	–	–	–	0.54
12	2-Cyclopenten-1-one, 3-methyl-	C_6_H_8_O	–	1.13	0.51	0.56	0.51
13	2-Cyclopenten-1-one, 2,3-dimethyl-	C_7_H_10_O	–	0.78	–	–	0.32
14	1,2-Ethanediol	C_2_H_6_O_2_	0.28	0.94	–	0.68	0.40
15	2-Furanmethanol	C_5_H_6_O_2_	0.68	0.64	0.97	0.95	0.81
16	Butanoic acid	C_4_H_8_O_2_	0.34	–	–	–	–
17	2(3H)-Furanone, dihydro-	C_4_H_6_O_3_	0.52	0.51	0.75	0.51	0.73
18	Naphthalene	C_10_H_8_	–	–	0.58	–	–
19	Azulene	C_10_H_8_	–	1.40	–	–	0.98
20	3,5-Dimethyl cyclopentenolone	C_7_H_10_O_2_	–	1.11	–	–	–
21	2-Cyclopenten-1-one, 2-hydroxy-3-methyl-	C_6_H_8_O_2_	1.14	0.94	0.45	2.10	1.62
22	Phenol, 2-methoxy-	C_10_H_12_O_2_	1.54	3.35	1.93	2.16	2.26
23	Naphthalene, 2-methyl-	C_11_H_10_	–	0.94	–	–	0.35
24	2-Cyclopenten-1-one, 3-ethyl-2-hydroxy-	C_7_H_10_O_2_	–	–	–	–	0.40
25	Phenol, 2-methoxy-4-methyl-	C_8_H_10_O_2_	0.58	1.23	0.83	1.09	1.19
26	Phenol, 2-methyl-	C_7_H_8_O	0.45	2.65	0.90	1.08	1.04
27	Phenol	C_6_H_6_O	1.65	6.95	3.17	3.26	3.46
28	Phenol, 4-ethyl-2-methoxy-	C_9_H_12_O_2_	0.40	–	0.78	1.01	–
29	1,1′-Biphenyl	C_12_H_10_	–	–	–	–	0.40
30	Benzeneethanol, 2-methoxy-	C_9_H_12_O_2_	–	1.59	–	–	1.09
31	Phenol, 2-ethyl-	C_8_H_10_O	–	1.41	–	–	0.31
32	Phenol, 2,4-dimethyl-	C_8_H_10_O	–	2.49	–	0.50	0.40
33	Phenol, 4-methyl-	C_7_H_8_O	0.50	3.04	1.13	1.27	1.05
34	Benzenemethanol	C_7_H_8_O	–	2.67	1.13	1.33	1.26
35	Phenol, 3-methyl-	C_9_H_12_O	0.41	–	–	–	–
36	Phenol, 4-(1-methylethyl)-	C_8_H_10_O	–	0.63	–	–	–
37	Phenol, 4-ethyl-	C_8_H_10_O	0.72	4.97	1.71	2.14	1.89
38	Phenol, 3-ethyl-	C_12_H_8_	–	0.71	0.48	0.59	0.44
39	Biphenylene	C_12_H_8_	–	0.87	–	–	0.42
40	Cyclopenta[de]naphthalene	C_12_H_8_	–	–	0.60	–	–
41	Acenaphthylene	C_12_H_8_	–	–	–	0.69	–
42	Phenol, 3-ethyl-5-methyl-	C_9_H_10_O_2_	–	0.55	–	–	–
43	2-Methoxy-4-vinylphenol	C_9_H_10_O_2_	1.29	1.29	1.33	4.11	0.73
44	Phenol, 2,6-dimethoxy-	C_8_H_10_O_3_	0.96	2.14	1.26	1.71	1.25
45	4-Methyl-syringol	C_9_H_12_O_3_	–	0.58	–	–	0.48
46	Phenol, 2-methoxy-4-(1-propenyl)-	C_10_H_12_O_2_	–	0.75	0.79	1.06	0.53
47	9H-Fluorene	C_13_H_10_	–	0.47	–	–	–
48	4-vinylphenol	C_8_H_8_O	1.39	3.26	2.05	4.90	1.10
49	2,3,5-Trimethoxytoluene	C_10_H_14_O_3_	–	0.99	–	–	–
50	1,4:3,6-Dianhydro-alpha-d-glucopyranose	C_6_H_8_O_4_	0.63	–	–	–	–
51	2-Furancarboxaldehyde, 5-(hydroxymethyl)	C_6_H_6_O_3_	0.64	–	–	–	0.54
52	Vanillin	C_8_H_8_O_3_	1.12	–	0.72	–	0.91
	**Total bio-oil yield (wt.%)**		30.38	28.97	36.24	31.49	32.28

#### Catalytic Effects on Bio-Oil Acidity and Stability

In comparison to the control SG sample, all catalysts reduced the percentage of undesired and unstable acid compounds. 30KP decreased acid compounds by 76%, leading to an increase of bio-oil pH value as reported by Mohamed et al. (2016b). Adding K_3_PO_4_ to bentonite or clinoptilolite increased the percentage of phenolic and aromatic compounds, compared to the bio-oil produced from 30Clino. 10KP/10Bento reduced acid compounds by 45% compared to SG, which is higher than the reduction of bio-oil acidity from 30Clino and 10KP/10Clino (27 and 24%, respectively). All the catalysts eliminated oxygenated compounds, including aldehydes and anhydrosugars. In addition, 30KP and 10KP/10Bento reduced furan compounds by up to 76 and 52%, respectively, in comparison with the control SG. The low acidity and oxygen content in bio-oil will further improve its stability and increase its energy density.

**FIGURE 3:**
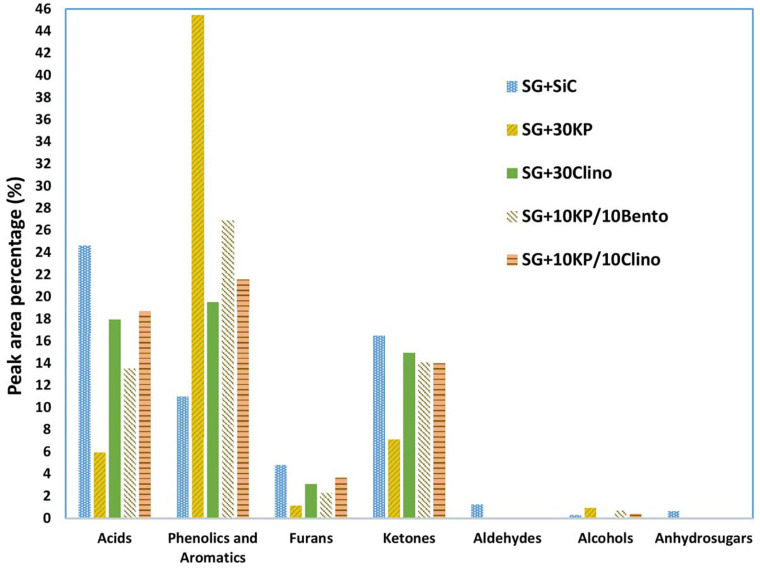
Lumped product concentrations in bio-oil (peak area %) produced from switchgrass mixed with SiC (control sample) and switchgrass mixed with different catalysts under microwave-assisted pyrolysis.

Figure 3 shows that both 30KP and 10KP/10Bento increased the phenolics and aromatic compounds by 309 and 144%, respectively, in comparison to the control. It is also seen that both 10KP/10Bento and 30KP eliminated vanillin from the bio-oil (see Table 8), which is one of the major oxygen-containing compounds contributing to bio-oil instability (Zabeti et al., 2012; Oh et al., 2017). Vanillin, a phenolic compound with the aldehyde group, is one of the most unstable and very reactive oxygen-containing groups and possesses low chemical stability compared to other phenolics groups and reduces bio-oil stability (Zabeti et al., 2012; Oh et al., 2017). Thus, eliminating those reactive compounds, including furfural and vanillin, can improve bio-oil chemical stability. It is seen that furfural was also reduced by 73% by 30KP and 10KP/10Bento. This confirms that catalyst mixtures can further improve the quality and stability of bio-oil compared to the single catalysts.

Wan et al. (2009) tested several inorganic chemical compounds (i.e., Al_2_O_3_, MgCl_2_, CoCl_2_, ZnCl_2_, Na_2_HPO_4_, K_2_Cr_2_O_7_, KAc, and AlCl_3_) for increasing microwave heating and improving the quality of bio-oil produced from corn stover and aspen under microwave pyrolysis. It was found that the chloride salts particularly affect the selectivity of bio-oil chemical compositions by producing more furan derivatives, and MgCl_2_ produced bio-oil rich in furfural with peak area of 80% (Wan et al., 2009). However, furan derivatives, aldehydes, and organic acids negatively affect bio-oil stability during storage in which density, viscosity, and calorific value can be dramatically affected (Mortensen et al., 2011; Oh et al., 2017; State et al., 2019; Sun et al., 2020).

#### Catalytic Effects on Bio-Oil Energy Density and Selectivity

**FIGURE 4:**
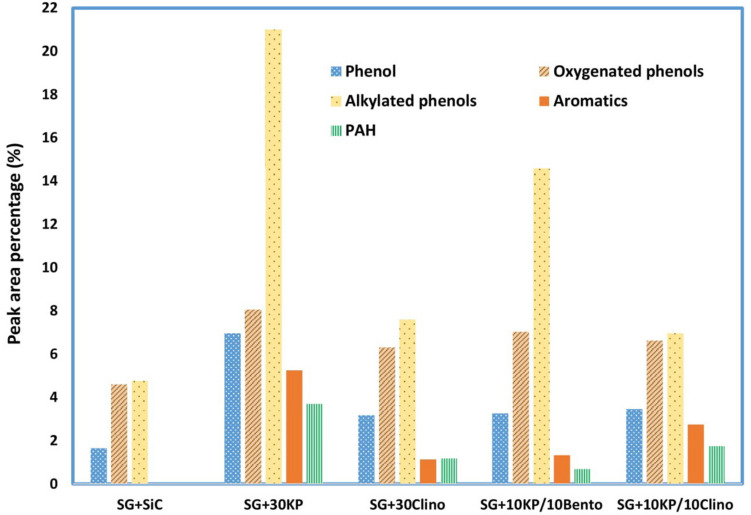
Effects of different catalysts and catalyst mixtures on concentrations of major compounds (acids, phenolics and aromatics, furans, ketones, aldehydes, alcohols, and anhydrosugars) in bio-oil under microwave-assisted pyrolysis, compared to the bio-oil produced from the control (switchgrass mixed with SiC).

Due to the high oxygen content of bio-oil (typically 35–40% by weight), bio-oil has a much lower energy density than liquid fossil fuels. Acetic acid not only increases bio-oil acidity, but also reduces the heating value since acetic acid contains about 53% oxygen. Thus, reducing acetic acid content not only reduces the acidity of bio-oil but also increases the heating value and improves bio-oil stability. In addition, all the catalysts increased the yield of phenolics and other aromatic compounds, with the highest yield achieved by 30KP and 10KP/10Bento. On the other hand, some hemicellulose-derived compounds, including aldehydes, were eliminated completely, as shown in Figure 4. All catalysts increased the yield of alkylated phenols, compared to the control. The highest yields were found for 30KP and 10KP/10Bento in which the yield of alkylated phenols was 341 and 207% higher than that of the control SG, respectively. The alkylated phenols are more favorable as a liquid fuel because they possess a high octane number and high heating values. Methyl phenol and phenol, for example, have a HHV of 34.15 and 33.11 MJ/kg, respectively (Zabeti et al., 2012). The bio-oil composition data indicated that the catalysts have primarily catalyzed lignin degradation and tar cracking.

Aromatic hydrocarbons, which have higher octane numbers, are particularly attractive chemicals that could be used as octane enhancers in gasoline (Chandrasekaran and Hopke, 2012; Rezaei et al., 2014). While 30KP and 10KP/10Clino showed the highest percentage of aromatics, 30KP showed the highest percentage of polycyclic aromatic hydrocarbons (PAH). In addition, 10KP/10Bento showed the lowest percentage of PAH compared to other catalysts, and no PAH was found for the control. The generation of PAH compounds by condensation and polymerization that leads to coke formation is a major challenge caused by catalyst deactivation (Rezaei et al., 2014; Kelkar et al., 2015). The increase in the production of PAH is probably a result of H-abstraction reactions that activates the aromatic molecules, and the introduction of acetylene propagates PAH formation by cyclization (Morf et al., 2002).

Although 30KP showed the highest percentages for all major compounds including aromatics and alkylated phenols, it increased the percentage of PAH compounds remarkably and increased the solid yield by more than 50% as a result of inhibiting hemicellulose decomposition and promoting coke deposition, which, in turn, lowered the microwave heating compared to the control and other catalyst mixtures. On the other hand, the catalyst mixtures improved the catalytic performance significantly by increasing the microwave heating rates, and 10KP/10Bento showed the highest heating rate in microwave catalytic pyrolysis, reduced solid yield, and lowered coke deposition remarkably in comparison with 30KP (Mohamed et al., 2019a). Catalyst mixtures also reduced the catalyst loads, which, in turn, will reduce the production cost of bio-oil and biochar from microwave catalytic pyrolysis. Future research is therefore needed to further optimize the performance for catalyst mixtures by changing the combinations of K_3_PO_4_ and clinoptilolite or bentonite using different loads. In addition, other low-cost natural catalysts with high microwave absorption ability should be examined.

In general, data from the literature agree with our findings. A study on bio-oil chemical composition showed that all catalysts catalyzed the cracking of vapors released from lignin decomposition, as reflected in increased conversion rates (Rajić et al., 2013). The lignin decomposition was shifted to a lower temperature (Rajić et al., 2013) that decreased pseudo-lignin activation energy. Clinoptilolite has been reported to catalyze the decomposition of lignin, yielding a bio-oil rich in phenolic compounds, and the amount of alkylphenols, pyrocatechols, and BTEXs (benzene, toluene, ethylbenzene and xylenes) generated from lignin decomposition also increased by clinoptilolite (Lee et al., 2016). Potassium was shown to prevent the production of acids, anhydrosugars, acetol, and xylose, while K_3_PO_4_ promotes hemicellulose char, gas, and H_2_O (Bulushev and Ross, 2011; Le Brech et al., 2016; Zhang et al., 2017), which is in line with the present study. Furthermore, K_3_PO_4_ mostly inhibited the primary hemicellulose oxygenates but mostly catalyzed lignin, as confirmed by the considerable increase in phenolic derivatives (Lu et al., 2013; Le Brech et al., 2016; Zhang et al., 2017).

From our previous studies, 10KP/10Bento gave the highest microwave heating rate (271°C/min), producing bio-oil with the lowest water content and viscosity, as well as biochar with the highest specific surface area and cation exchange capacity (CEC) (Mohamed et al., 2016a, b). In addition, it was found that the biochar produced from 10KP/10Bento showed superior performance on reducing toxicity and the uptake of heavy metals (i.e., Pb, Ni, and Co) and also increasing plant growth in soils contaminated by heavy metals (Mohamed et al., 2017). The use of catalysts during catalytic microwave pyrolysis of switchgrass produced biochars containing significant amounts of plant nutrients, and the engineered biochars can be used to remediate and reclaim contaminated soils by decreasing phytotoxicity of heavy metals, enhancing soil fertility and increasing crop yield with reduction in groundwater contamination. Therefore, there is no need to separate catalysts from the produced biochars if they are targeted for soil applications. Thus, it can be concluded that catalyst mixtures such as K_3_PO_4_ and bentonite tested in this study would be a much better choice than single catalysts for microwave pyrolysis for the production of high-quality bio-oil and biochar.

## Conclusion

The synergistic effects of catalyst mixtures on biomass decomposition were examined at similar heating rates in a TGA. The activation energy for 10KP/10Bento and 10KP/10Clino (10/10 wt.%) was slightly lower than, or similar to, other single catalysts at 30 wt.% load. In addition, catalyst mixtures increased mass loss rate, reduced activation energy, and increased microwave heating rate markedly when compared to the case of single catalyst at a catalyst load of 30 wt.%. Acids, aldehydes, anhydrosugars, and other reactive oxygen-containing compounds, such as furfural and vanillin, in bio-oil were remarkably reduced or eliminated, while phenolics and aromatics increased with the addition of catalysts. All catalysts increased the concentration of alkylated phenols compared to the control, with the highest values found for 30KP and 10KP/10Bento. Those findings suggest that the performance of the microwave-assisted catalytic pyrolysis reactor can be improved by using catalyst mixtures, which, in turn, leads to improved stability and selectivity of bio-oil and reduced activation energy, at a load lower than single catalysts.

## Data Availability Statement

The datasets presented in this study can be found in online repositories. The names of the repository/repositories and accession number(s) can be found in the article/Supplementary Material.

## Author Contributions

BM carried out all the experimental work, data analysis, and manuscript preparation. NE, CK, and XB provided the comments and feedback and edited the manuscript. All the authors read and approved the final manuscript.

## Conflict of Interest

The authors declare that the research was conducted in the absence of any commercial or financial relationships that could be construed as a potential conflict of interest.
