# Supplementary material for: Synergistic Effects of Catalyst Mixtures on Biomass Catalytic Pyrolysis
Source: Front Bioeng Biotechnol. 2020 Dec 14;8:615134. doi: 10.3389/fbioe.2020.615134 (PMC7767908; doi:10.3389/fbioe.2020.615134)
Supplement: Supplementary file 1 [file Data_Sheet_1.docx]

**Synergistic Effects of Catalyst Mixtures on Biomass Catalytic Pyrolysis**

**Badr A. Mohamed ^1,2^, Naoko Ellis ^2^, Chang Soo Kim ^2,3^ and Xiaotao Bi ^2,^ ⃰**

^1^ Department of Agricultural Engineering, Cairo University, Giza, Egypt

^2^ Department of Chemical and Biological Engineering, University of British Columbia,

Vancouver, BC V6T 1Z3, Canada

^3^ Clean Energy Research Center, Korea Institute of Science and Technology,

14 gil 5 Hwarang-no Seongbuk-gu Seoul Korea 136-791

⃰ Corresponding author: Prof. Dr. Xiaotao Bi

Tel: 1-604-822-4408; Fax: 1-604-822-6003; Email: [tony.bi@ubc.ca](mailto:tony.bi@ubc.ca)

**Supplementary Material**


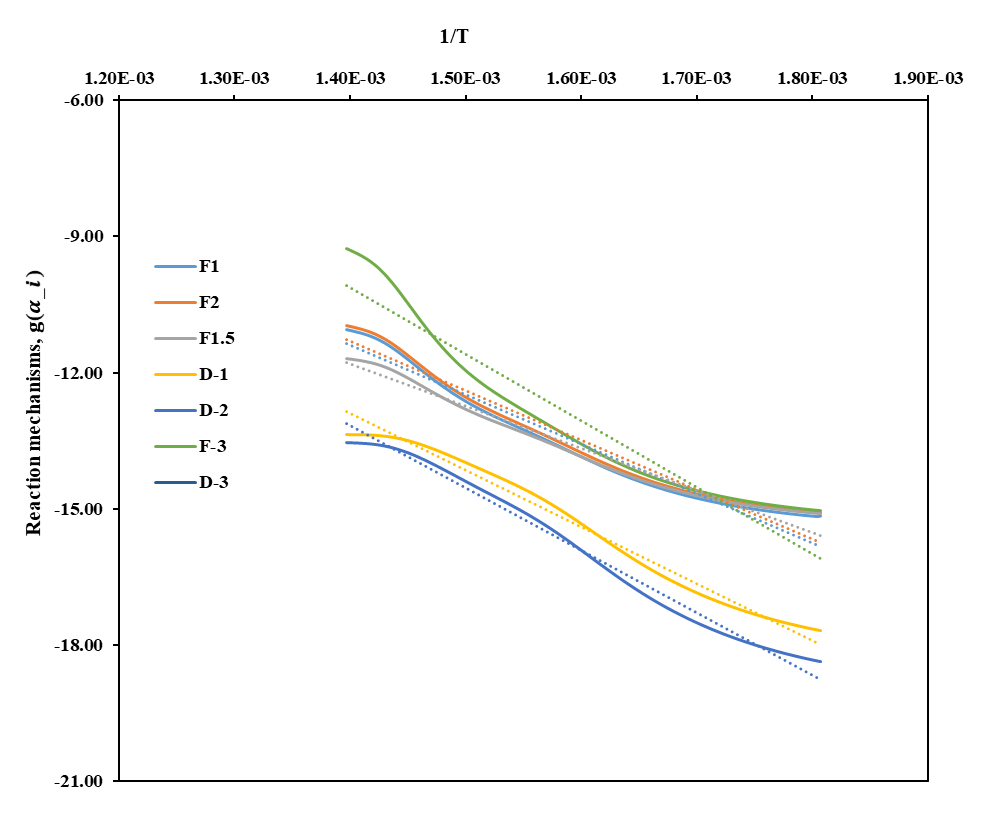


**Figure S1. The regression for different kinetic reaction mechanisms for switchgrass without catalyst at the first stage (208-450°C) and 100 °C/min.**


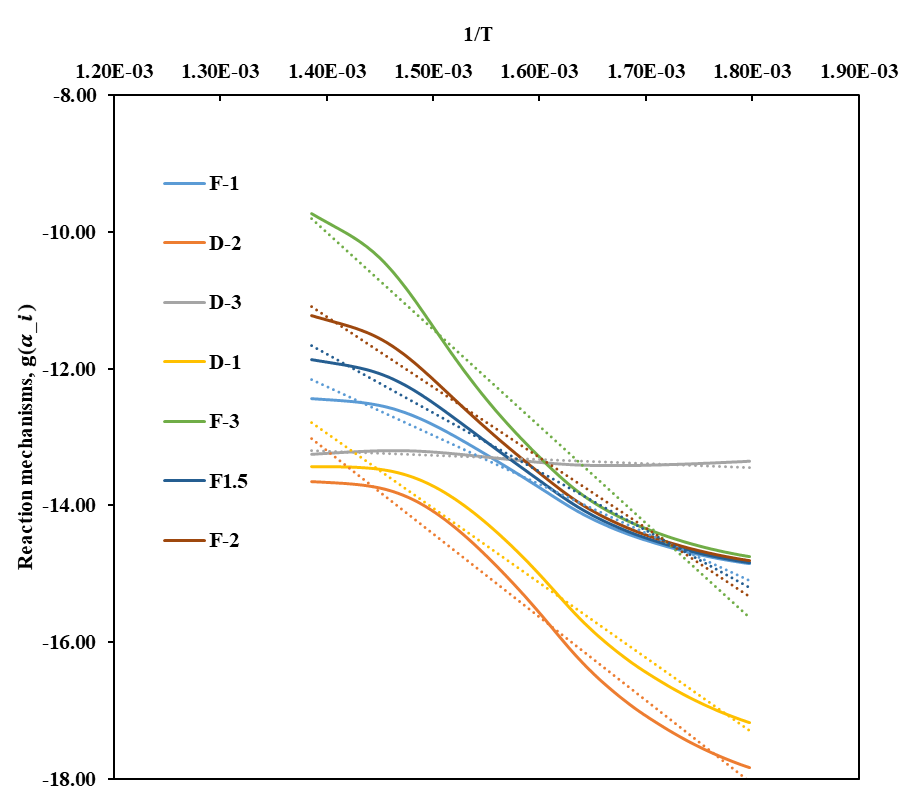


**Figure S2. The regression for different kinetic reaction mechanisms for switchgrass mixed with 30 wt.% K_3_PO_4_ (30KP) at the first stage (208-450°C) and 100 °C/min.**


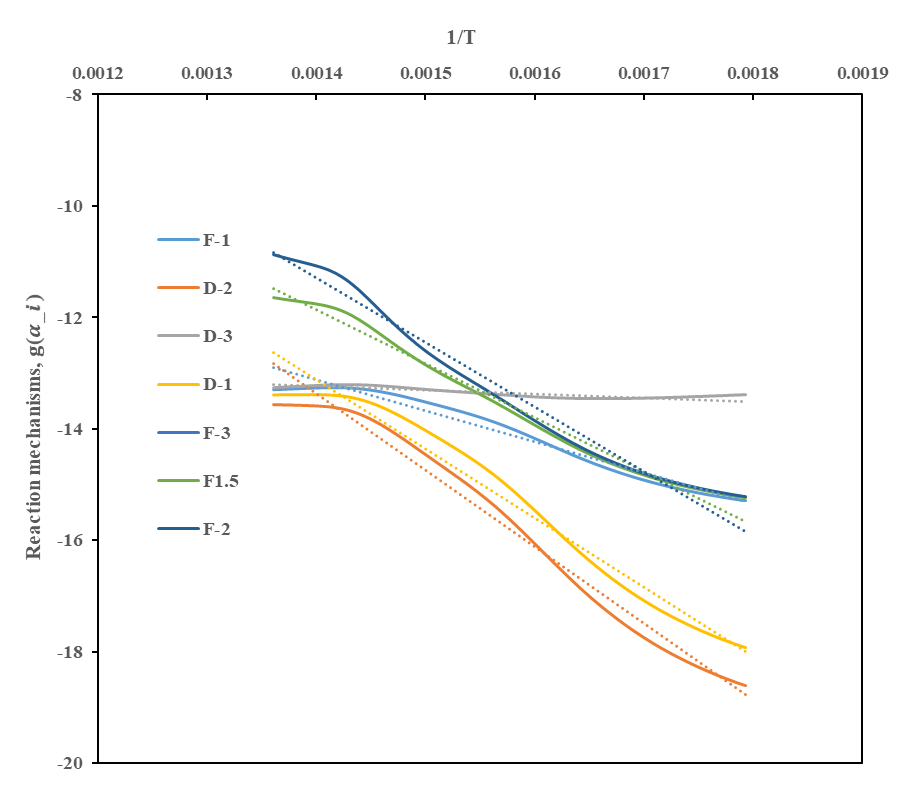


**Figure S3. The regression for different kinetic reaction mechanisms for switchgrass mixed with 30 wt.% Bentonite (30Bento) at the first stage (208-450°C) and 100 °C/min.**


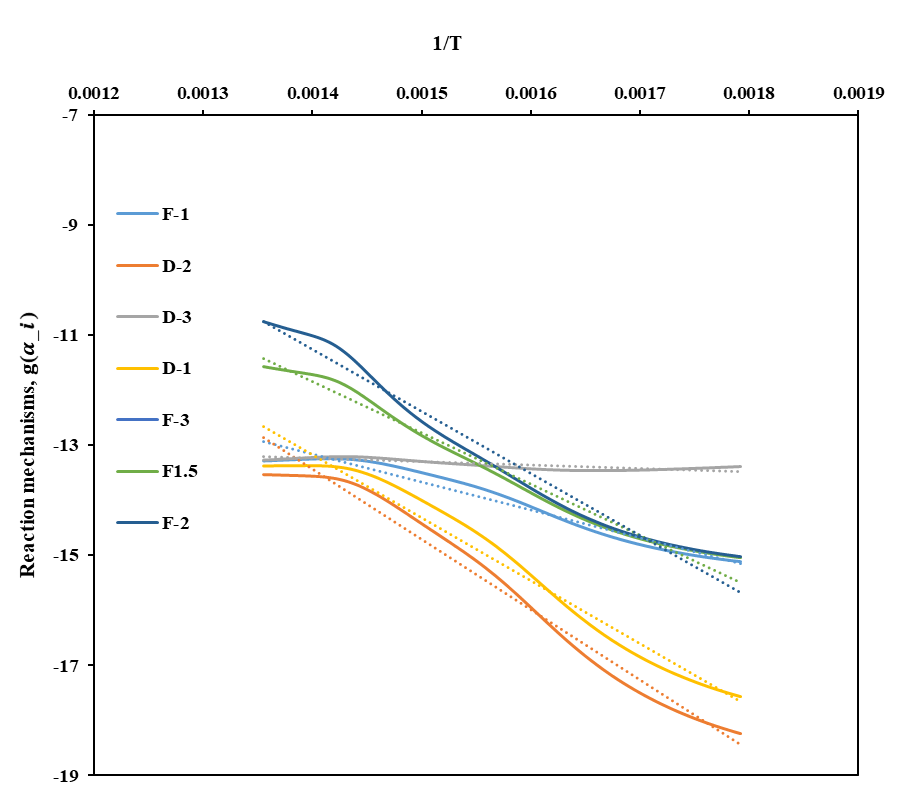


**Figure S4. The regression for different kinetic reaction mechanisms for switchgrass mixed with 30 wt.% Clinoptilolite (30Clino) at the first stage (208-450°C) and 100 °C/min.**


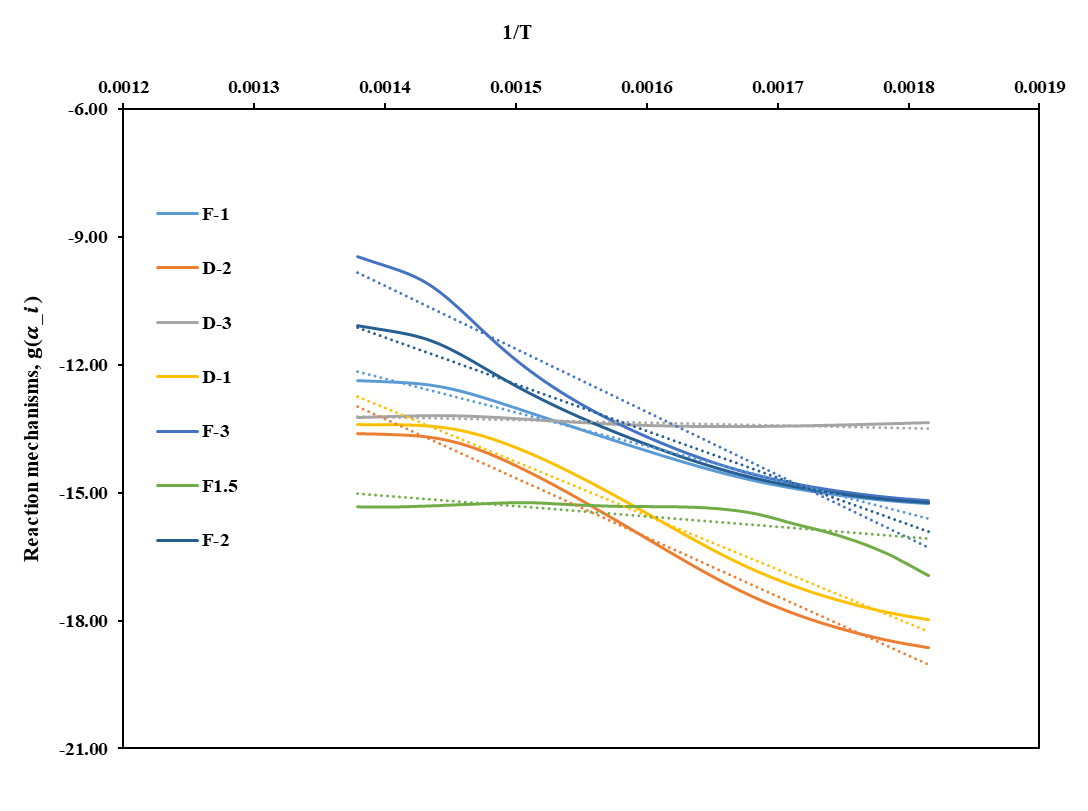


**Figure S5. The regression for different kinetic reaction mechanisms for switchgrass mixed with 10 wt.% K_3_PO_4_ + 10 wt.% Bentonite (10KP/10Bento) at the first stage (208-450°C) and 100 °C/min.**


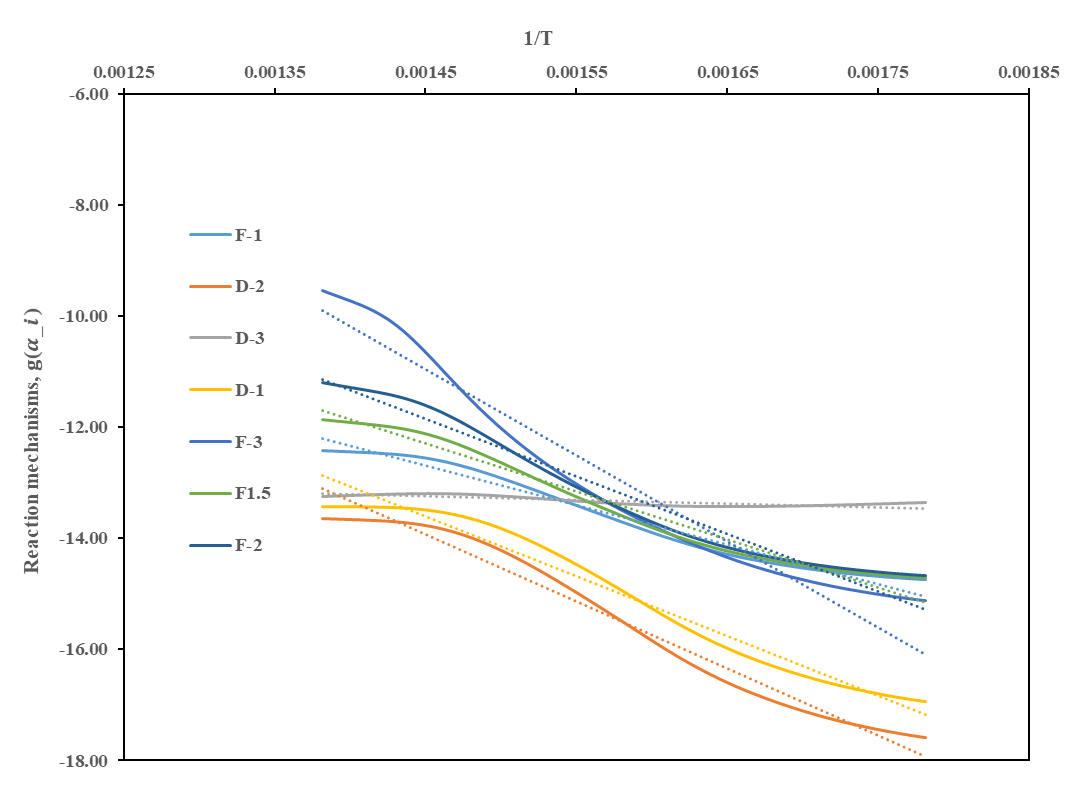


**Figure S6. The regression for different kinetic reaction mechanisms for switchgrass mixed with 10 wt.% K_3_PO_4_ + 10 wt.% Clinoptilolite (10KP/10Clino) at the first stage (208-450°C) and 100 °C/min.**
